# Neighborhood and weight-related health behaviors in the Look AHEAD (Action for Health in Diabetes) Study

**DOI:** 10.1186/1471-2458-10-312

**Published:** 2010-06-04

**Authors:** Tiffany L Gary-Webb, Kesha Baptiste-Roberts, Luu Pham, Jacqueline Wesche-Thobaben, Jennifer Patricio, F Xavier Pi-Sunyer, Arleen F Brown, LaShanda Jones, Frederick L Brancati

**Affiliations:** 1 Department of Epidemiology, Columbia Mailman School of Public Health, New York, NY, USA; 2 Department of Epidemiology, Johns Hopkins Bloomberg School of Public Health, Baltimore, MD, USA; 3 Division of General Internal Medicine, Johns Hopkins School of Medicine, Baltimore, MD, USA; 4 Department of Biostatistics, Johns Hopkins Bloomberg School of Public Health, Baltimore, MD, USA; 5 Division of Internal Medicine, University of Pittsburgh, Pittsburgh, PA, USA; 6 Department of Medicine, St. Luke's—Roosevelt Hospital Center, New York, NY, USA; 7 Division of General Internal Medicine and Health Services Research, Department of Medicine, David Geffen School of Medicine at UCLA, Los Angeles, CA, USA; 8 Department of Psychiatry, University of Pennsylvania, Philadelphia, PA, USA

## Abstract

**Background:**

Previous studies have shown that neighborhood factors are associated with obesity, but few studies have evaluated the association with weight control behaviors. This study aims to conduct a multi-level analysis to examine the relationship between neighborhood SES and weight-related health behaviors.

**Methods:**

In this ancillary study to Look AHEAD (Action for Health in Diabetes) a trial of long-term weight loss among individuals with type 2 diabetes, individual-level data on 1219 participants from 4 clinic sites at baseline were linked to neighborhood-level data at the tract level from the 2000 US Census and other databases. Neighborhood variables included SES (% living below the federal poverty level) and the availability of food stores, convenience stores, and restaurants. Dependent variables included BMI, eating patterns, weight control behaviors and resource use related to food and physical activity. Multi-level models were used to account for individual-level SES and potential confounders.

**Results:**

The availability of restaurants was related to several eating and weight control behaviors. Compared to their counterparts in neighborhoods with fewer restaurants, participants in neighborhoods with more restaurants were more likely to eat breakfast (prevalence Ratio [PR] 1.29 95% CI: 1.01-1.62) and lunch (PR = 1.19, 1.04-1.36) at non-fast food restaurants. They were less likely to be attempting weight loss (OR = 0.93, 0.89-0.97) but more likely to engage in weight control behaviors for food and physical activity, respectively, than those who lived in neighborhoods with fewer restaurants. In contrast, neighborhood SES had little association with weight control behaviors.

**Conclusion:**

In this selected group of weight loss trial participants, restaurant availability was associated with some weight control practices, but neighborhood SES was not. Future studies should give attention to other populations and to evaluating various aspects of the physical and social environment with weight control practices.

## Background

It is estimated that 97 million adults in the United States are either overweight or obese [1]. Obesity, defined as a body mass index (BMI) of 30 kg/m^2 ^or higher in adults, is a complex disease that arises from interactions between multiple genes, as well as behavioral and environmental factors [1]. Furthermore, obesity is a serious risk factor for many chronic conditions (diabetes, hypertension, hypercholesterolemia, stroke, heart disease, certain cancers, and arthritis) and has been reported to markedly decrease life expectancy [2-4]. The prevalence of obesity was relatively stable between 1960 and 1980, but has dramatically increased over the past 20 years [5]. Although the health risks of obesity are well established, there is less certainty about the management of the disease. Lifestyle modification programs to address obesity prevention and weight loss have achieved only moderate success, particularly interventions for long-term weight loss [6].

New public health approaches to the obesity problem are urgently required. One factor that may play a role in the risk of obesity is the neighborhood environment [7,8]. Neighborhood socioeconomic conditions are known to affect health even after controlling for individual-level socio-demographic factors [9-11]. Recent data suggests that the neighborhood environment may influence risk of chronic diseases such as cardiovascular disease, type 2 diabetes, and related health behaviors such as decreased levels of physical activity [12-22]. A few studies have demonstrated that living in neighborhoods with low socio-economic status (SES) is associated with an increased risk of obesity [23,24]. However, there are few data on neighborhood and weight-control behaviors. Investigating novel correlates of weight control is necessary given there is overwhelming evidence showing that weight loss is associated with marked improvement in health status, particularly, blood pressure and glucose control [25-27].

Therefore, we conducted a multi-level analysis to examine the relationship between neighborhood SES and weight-related variables at baseline among overweight participants with type 2 diabetes enrolled in the Look AHEAD study. We hypothesized that: 1) poorer neighborhood SES would be associated with poorer eating patterns and weight control behaviors independent of individual-level socio-economic status and 2) more availability of stores with healthy options (i.e. food stores) in the neighborhood would be associated with better eating patterns and weight control behaviors independent of individual-level socio-economic status.

## Methods

### Study Population of the Parent Study

The primary objective of the Look AHEAD study [28] is to examine, in overweight volunteers with type 2 diabetes, the long-term effects of an intensive lifestyle intervention program designed to achieve and maintain weight loss by decreased caloric intake and increased physical activity. The intervention group is compared to a control condition involving a program of diabetes education and support. The primary basis for the comparison is the incidence of serious cardiovascular events. Other outcomes, including cardiovascular disease risk factors, diabetes-related metabolic factors and complications, and the cost-effectiveness of the intensive intervention are also studied. Participants are 5,145 volunteers with type 2 diabetes who are 45-75 years of age and overweight or obese (body mass index [BMI] ≥ 25 kg/m^2^).

### Study Population of the Ancillary Study

This ancillary study was conducted at baseline using Look AHEAD participants at 4 clinical sites; Baltimore(n = 302), Philadelphia(n = 293), Pittsburgh(n = 321), and New York(n = 303). Sites were chosen because of their close geographic proximity and similar demographic profile. The total study sample for this ancillary consists of 1219 participants with complete data on neighborhood environment. Addresses were used to identify the corresponding census tracts for each participant (neighborhood) as defined by the 2000 Census using a process called geocoding and software program ArcGIS™. The program matches imported addresses to geographic maps and other geographic data. Matches are rated with scores from 0 (no match) to 100 (perfect match); we accepted matches with 80% certainty or more. Once we identified the census tracts and corresponding data for each participant, these data were linked to the individual-level data collected during the Look AHEAD trial. A description of all of the main variables used in this analysis is summarized in Table 1.

**Table 1:** Selected Characteristics of 1219 Look AHEAD participants

Characteristics	
**Socio-demographic Characteristics**	
Age (years)	59.5 ± 6.7
Sex	
Male	501 (41.1)
Education (years)^†^	
16+	192 (16.2)
13 - 16	389 (32.7)
≤ 12	607 (51.1)
Race	
Black	327 (26.8)
White	795 (65.2)
Other	97 (8.0)
Income ^‡^	
< $20,000	79 (7.5)
$20,000-$40,000	184 (17.6)
$40,000-$60,000	216 (20.7)
$60,000-$80,000	185 (17.7)
≥ $80,000	382 (36.5)
Body mass index [BMI, kg/m^2^]	36.1 ± 5.7
Overweight [20-29.9]	163 (13.4)
Obese [30-34.9]	780(64.0)
Extreme Obesity[≥ 35]	276 (22.6)
	
**Neighborhood Census Tract Indicators**	
Percent Below Poverty [Range = 0, 0.67]	0.11 ± 0.10
Cubbin Deprivation Score [Range = -2.9, 2.8]	0.001 ± 0.78
Diez-Roux Deprivation Score [Range = -18.7, 12.0]	0.010 ± 5.0
Food Stores [Range = 0, 9]	1.3 ± 1.5
Convenience Stores [Range = 0, 4]	0.6 ± 0.8
Restaurants [Range = 0, 104]	6.8 ± 10.8
Other Food Stores [Range = 0, 9]	2.0 ± 2.8
Other Food Service [Range = 0, 9]	2.6 ± 3.2
	
**Dietary Intake (daily)**	
Total Fat (%) ^ξ^	
≥ 35	465 (73.9)
< 35	164 (26.1)
Saturated Fat (%) ^ξ^	
≥ 10	519 (82.5)
< 10	110 (17.5)
Fruit & Vegetable (servings) ^ξ^	
< 9	584 (92.8)
≥ 9	45 (7.2)
	
**Eating Patterns**	
Eat breakfast (days/week)^§^	
7 vs < 7	839 (69.1)
Eat breakfast at a fast food restaurant^€^	
≥1 vs. 0	237 (27.7)
Eat breakfast at non-fast food restaurant^ψ^	
≥1 vs 0	335(27.7)
Eat lunch (days/week)^¥^	
7 vs < 7	827 (68.1)
Each lunch at a fast food restaurant^χ^	
≥1 vs 0	440 (36.4)
Eat lunch at a non-fast food restaurant^χ^	
≥1 vs 0	618 (51.2)
Eat dinner (days/week)^¢^	
7 vs < 7	1081 (89.2)
Eat dinner at a fast food restaurant^χ^	
≥1 vs 0	285 (23.6)
Eat dinner at a non-fast food^τ^	
≥1 vs 0	876 (72.5)
	
**Weight Loss**	
Attempting weight loss^§^	
Yes	1161 (95.6)
Participation in weight loss program ^¥^	
Yes	748 (61.6)
	
**Weight Loss Control Practices (yes/no)**	
***Food***	
Count fat grams^¥^	194 (16.0)
Cut out between meal snacking ^τ^	428 (35.4)
Eat less high carbohydrate foods^€^	713 (58.9)
Reduce the number of calories eaten^ά^	661 (54.5)
Record what you eat daily ^γ^	442 (36.4)
Decrease fat intake ^τ^	636 (52.6)
Eat meal replacements	193 (15.9)
Cut out sweets and junk food from diet	763 (63.0)
Increase fruit and vegetables	789 (65.3)
Fast or go without the food entirely^ά^	74 (6.1)
Count calories^ά^	193 (15.9)
Eat special low calorie diet foods	256 (21.2)
Drink fewer alcoholic beverages	222 (18.5)
Eat less meat^€^	430 (35.5)
Weight control food outcome summary score [0-13]	4.92 ± 3.06
***Physical Activity***	
Keep a graph of exercise^¥^	87 (7.2)
Increase your exercise levels^ά^	671 (55.3)
Use home exercise equipment^§^	374 (30.8)
Record exercise daily^¥^	149 (12.3)
Weight control physical activity outcome summary score [0-4]	1.05 ± 0.99
***Weight Control***	
Keep graph of weight^§^	82 (6.8)
Go to a weight loss group^§^	173 (14.2)
Take diet pills^§^	55 (4.5)
Other weight control activities	111 (13.1)
Smoke cigarettes^§^	51 (4.2)
Weight control weight loss summary score [0-4]	0.38 ± 0.64
	
**Resource Use (yes/no)**	
***Food***	
Food preparation equipment purchased in the past year^§^	*621 (51.1)*
***Physical Activity***	
Positive feeling about exercise₫	765 (63.9)
Indoor exercise purchases in the past year ^γ^	411 (33.8)
Outdoor exercise purchases in the past year ^γ^	103 (8.5)
Gym membership purchase in the past year ^ή^	265 (21.7)
Resource use physical activity outcomes summary score	1.27 ± 0.96
***Weight Control***	
Class membership/services for weight loss purchases in the past year^§^	398 (32.8)
Joined a weight loss program in the past year^ά^	161 (13.3)
Resource use weight control outcomes summary score	0.46 ± 0.61

^†^N = 1188, ^‡^N = 1046, ^ξ^N = 629, ^§^N = 1215, ^¥^N = 1214, ^¢^N = 1212, ^€^N = 1210, ^ψ^N = 1211, ^χ^N = 1208, ^τ^N = 1209, ^ℓ^n = 1203, ^Ω^N = 1199, ^Δ^N = 1205, ^∏^N = 1200, ^₫^N = 1197, ^ά^N = 1213, ^ή ^N = 1219, ^γ ^N = 1216

**All results are presented as n(%) or mean ± SD**

Higher Cubbin and Diez-Roux score= lower SES

### Main Data Sources

Data are derived from the 2000 US Census long form and include demographic characteristics (age, race, sex), housing characteristics (housing structure, number of rooms, telephone surface), economic characteristics (occupation, place of work and journey to work) and financial characteristics (value of home, rent, utilities cost) for each census tract.

We also used data from the 2004 Consumer Expenditure database which outlines the locality of food stores using a multi-level hierarchical classification system. The data are derived from an extensive modeling effort using the 1994, 1995, 1996, 1997, and 2000 Consumer Expenditure Survey data from the Bureau of Labor Statistics (BLS), in addition to the latest 1998 overview data. The BLS survey averages over 5,000 households four times a year using a rotating sampling frame. We used aggregate data at the census tract level (estimated at 3000-5000 persons).

Participants in the Look AHEAD study underwent extensive data collection at baseline, including interview, physical examination, and blood and urine assays[28]. Although the trial will last over 10 years, this manuscript is restricted to data collected at baseline only. The parent study was approved by the Johns Hopkins Western Institutional Review Board and all participants signed written informed consent to participate in the study.

### Key Independent Variables

Using the Census data, indices of neighborhood socio-economic status developed by Diez-Roux and Winkleby/Cubbin, were created using variables such as the % of persons living below poverty, % of adults with a college degree, median household income, % of persons earning interest income, % of adults in executive/managerial occupations, and % of adults who are unemployed. After considering these measures used in previous studies [29-31], we ultimately decided on the single item "% of individuals in the census tract living below the federal poverty line" because this measure is highly correlated with other census-based indices and has been shown to be similarly predictive of health outcomes [31].

Data on food availability in the census tract was categorized using the North American Industry Classification System (NAICS) definitions into: 1) ***food stores ***these establishments retail food and beverages merchandise from fixed point-of-sale locations. Establishments in this subsector have special equipment (e.g., freezers, refrigerated display cases, refrigerators) for displaying food and beverage goods. They have staff trained in the processing of food products to guarantee the proper storage and sanitary conditions required by regulatory authority; includes grocery stores and supermarkets; 2) ***convenience stores ***these establishments primarily engaged in retailing a limited line of goods that generally includes milk, bread, soda, and snacks; and 3) ***restaurants ***these establishments primarily engaged in providing food services to patrons who order and are served while seated (i.e. waiter/waitress service and pay after eating. they may provide this type of food service to patrons in combination with selling alcoholic beverages, providing carry out services, or presenting live non-theatrical entertainment; includes full-service, fast food, and carryout.

### Key Dependent Variables

Dependent variables included eating patterns, weight loss control practices, and BMI. Variables capturing participant eating patterns consisted of reports of eating breakfast, lunch, and dinner and whether they ate at fast-food or non-fast food restaurants. Dietary intake, daily total fat, saturated fat, and fruit and vegetable intake was measured on a sub-set of individuals at baseline (629 participants in the ancillary study). Questions related to attempting weight loss and participating in weight loss programs and weight loss control practices related to food, physical activity, and weight control resource use were also examined. Examples of resources use include purchases of exercise equipment or weight loss program membership. For multivariate analysis purposes, individual questions were summarized to create separate scores for resource use related to physical activity, weight control, and food preparation. BMI was calculated using measured height and weight.

### Statistical Analysis

In this analysis, the main independent variables were the neighborhood factors and the main dependent variables were individual-level weight-related variables from the Look AHEAD study. Descriptive statistics were used to describe the study population.

Multi-level analyses were used to analyze the aggregate and individual level data [32-34]. Recognizing that when studying group-level variables, individuals are nested within those groups, multi-level analyses are designed to account for this clustering. Specifically, the model building first identifies the most predictive set of individual-level variables. Then aggregate-level variables are added. At each level, all variables and their interaction effects are tested. Random effects terms are then added as additional parameters to account for extra area-level variability not explained by the model and included variables (overdispersion) [32].

In the current study, the association between neighborhood factors (% poverty, food availability for the census tract) and individual-level weight-related outcomes were determined while accounting for individual level SES (personal income and education). This enabled us to determine the independent effects of the neighborhood SES. Other potential confounders included in the models were: age, sex, and race. All analyses were conducted using STATA statistical software, version 9.

## Results

### Selected Baseline Characteristics of Study Participants

Selected baseline characteristics of the study participants are presented in Table 1. Participants were on average 59.5 ± 6.7 years of age, 41% male, and 27% were Black/African American. About half of participants had at least some college education; the majority of participants had annual incomes > $40,000. All participants were at least overweight or obese (BMI > 25 kg/m^2^),eligibility criteria for Look AHEAD.

Participant neighborhoods were diverse. Of all the neighborhoods represented in the study, the mean % of those living below the federal poverty level was 11%. Neighborhoods on average had 1.3 ± 1.5 food stores, 0.6 ± 0.8 convenience stores, and 6.8 ± 10.8 restaurants (fast food and non-fast food). Overall, there were 920 unique census tracts represented in the study; Baltimore = 201, New York= 257, Philadelphia = 245, Pittsburgh = 217. The number of participants per census tract ranged from 1-6.

With respect to dietary intake and eating patterns, most participants did not meet the recommended intakes for total fat, saturated fat, and fruit and vegetables. Most participants reported eating breakfast, lunch and dinner every day, and about a quarter reported eating those meals at a fast food restaurant more than once a week.

The vast majority of participants reported that they were currently attempting weight loss (96%) and 62% reported that they had participated in a weight loss program before the study. Many reported various weight control strategies such as cutting out sweets and junk food from their diets (63%), increasing fruit and vegetables (65%), and increasing exercise levels (55.3). With respect to resources spent on food, physical activity, and weight control, the most common were purchase of food preparation equipment (51%), indoor exercise equipment (33.8%), and class membership services for weight loss (33%).

### Association between Neighborhood and Eating Patterns

Table 2 outlines the association between neighborhood and eating patterns. Participants living in neighborhoods with more restaurantswere significantly more likely to eat breakfast and lunch at restaurants that were not fast food restaurants compared to those living in neighborhoods with fewer restaurants. Furthermore, they were significantly more likely to eat dinner 7 days per week.

**Table 2:** Prevalence Ratios and 95% confidence intervals for Neighborhood Indicators and Eating Patterns among 1219 participants in the Look AHEAD Study

Neighborhood Indicator		Eat breakfast (days/week)	Eat breakfast at a fast food restaurant (days/week)	Eat breakfast at restaurants that are not fast food restaurants (days/week)	Eat lunch (days/week)	Eat lunch at a fast food restaurant (days/week)	Eat lunch at restaurants that are not fast food restaurants (days/week)	Eat dinner (days/week)	Eat dinner at a fast food restaurant (days/week)	Eat dinner at restaurants that are not fast food restaurants (days/week)
Below Poverty (%)^†^	2	1.22 (0.98, 1.53)	0.93 (0.67, 1.28)	0.89 (0.70, 1.14)	0.99 (0.79, 1.24)	1.04 (0.86, 1.27)	0.96 (0.84, 1.10)	0.92 (0.71, 1.49)	1.17 (0.88, 1.54)	1.00 (0.92, 1.08)
	3	1.12 (0.88, 1.45)	1.11 (0.79, 1.55)	1.08 (0.83, 1.40)	1.17 (0.93, 1.48)	0.99 (0.80, 1.23)	1.03 (0.89, 1.20)	1.25 (0.77, 2.03)	1.13 (0.84, 1.53)	1.00 (0.92, 1.10)
										
Food Stores	≥1 vs <1	1.02 (0.85, 1.23)	1.22 (0.94, 1.10)	1.03 (0.84, 1.26)	1.20 (0.99, 1.44)	0.93 (0.79, 1.09)	1.00 (0.89, 1.11)	1.07 (0.73, 1.56)	1.06 (0.84, 1.33)	0.96 (0.89, 1.02)
										
Convenience Stores	≥1 vs <1	0.99 (0.82, 1.18)	**1.38** **(1.08, 1.77)**	1.15 (0.93, 1.63)	0.99 (0.83, 1.17)	1.06 (0.90, 1.24)	0.99 (0.84, 1.11)	1.29 (0.90, 1.84)	1.06 (0.86, 1.32)	0.94 (0.88, 1.01)
										
Restaurants^†^	2	1.07 (0.86, 1.33)	0.94 (0.69, 1.30)	1.11 (0.87, 1.43)	**1.24** **(1.00, 1.53)**	0.94 (0.29, 1.30)	1.08 (0.94, 1.25)	0.99 (0.62, 1.58)	0.86 (0.65, 1.11)	0.97 (0.88, 1.06)
	3	1.15 (0.94, 1.42)	1.27 (0.95, 1.70)	**1.28** **(1.01, 1.62)**	**1.24** **(1.00, 1.53)**	0.91 (0.75, 1.11)	**1.19** **(1.04, 1.36)**	**1.61** **(1.06, 2.45)**	1.01 (0.79, 1.29)	1.03 (0.95, 1.11)

All models adjusted for age, sex, education, income and race

^†^All represent comparisons to tertile 1 (reference groups)

### Association between Neighborhood and Weight Loss Control Practices

Neighborhood SES had little association with weight control practices (see Table 3). Those who lived in neighborhoods with more restaurants were less likely to be attempting weight loss, and more likely to participate in weight loss control practices related to food and physical activity than those who lived in neighborhoods with fewer restaurants. There was no significant association seen for neighborhood and BMI.

**Table 3:** Analyses for Neighborhood Indicators, and Weight Control and BMI among 1219 participants in the Look AHEAD Study

Neighborhood Indicator	Tertile	Weight Loss (yes/no) PR (95% CI)	Past Weight Loss Program Participation (yes/no) PR 95% CI	Weight Control Food Β coefficient (95% CI)	Weight Control Physical Activity Β coefficient (95% CI)	Weight Control Β coefficient (95% CI)	BMI (kg/m^2^) β coefficient (95% CI)
Below Poverty^† ^(%)	2	**0.88** **(0.80, 0.97)**	0.94 (0.86, 1.03)	-0.24 (-0.68, 0.19)	0.01 (-0.13, 0.16)	0.08 (-0.01, 0.18)	0.01 (-0.86, 0.86)
	3	**0.89** **(0.81, 0.98)**	1.02 (0.92, 1.12)	0.17 (-0.32, 0.67)	0.10 (-0.05, 0.25)	0.03 (-0.08, 0.13)	0.42 (-0.54, 1.39)
							
Food Stores	≥1 vs <1	**0.96** **(0.94, 0.99)**	0.98 (0.91, 1.06)	0.29 (-0.08, 0.67)	0.18 (0.06, 0.30)	0.10 (0.02, 0.18)	0.34 (-0.38, 1.07)
							
Convenience Stores	≥1 vs <1	0.99 (0.97, 1.02)	0.95 (0.88, 1.03)	-0.19 (-0.55, 0.16)	0.01 (-0.11, 0.13)	-0.04 (-0.12, 0.03)	-0.11 (-0.82, 0.61)
							
Restaurants^†^	2	**1.11** **(1.05, 1.17)**	1.06 (0.97, 1.17)	-0.03 (-0.47, 0.40)	0.06 (-0.08, 0.20)	0.004 (-0.09, 0.10)	-0.16 (-1.01, 0.70)
	3	**0.93** **(0.89, 0.97)**	1.00 (0.91, 1.09)	**0.42** **(0.005, 0.84)**	**0.24** **(0.09, 0.38)**	0.07 (-0.02, 0.17)	0.40 (-0.45, 1.26)

All models adjusted for age, sex, education, income and race

^†^All represent comparisons to tertile 1 (reference groups)

### Association between Neighborhood and Resource Use

Those living in neighborhoods with more poverty were significantly more likely to have purchased food preparation equipment in the past year compared to those living in neighborhoods with the least poverty (PR = 1.36, see Table 4).

**Table 4:** Analyses for Neighborhood Indicators, Weight Control Practices and Resource Use

Neighborhood Indicator	Tertile	Resource Use Physical Activity β coefficient (95% CI)	Resource Use Weight Control β coefficient (95% CI)	Food preparation equipment purchased in the past year (yes/no) PR (95% CI)
Below Poverty^† ^(%)	2	-0.08 (-0.23, 0.06)	0.05 (-0.04, 0.14)	1.09 (0.93, 1.28)
	3	0.02 (-0.14, 0.19)	0.04 (-0.07, 0.15)	**1.36** **(1.16, 1.59)**
				
Food Stores	≥1 vs <1	-0.06 (-0.18, 0.07)	0.03 (-0.04, 0.11)	1.03 (0.92, 1.17)
				
Convenience Stores	≥1 vs <1	-0.02 (-0.14, 0.10)	-0.04 (-0.12, 0.03)	0.90 (0.80, 1.02)
				
Restaurants^†^	2	0.06 (-0.09, 0.20)	-0.02 (-0.11, 0.07)	1.09 (0.96, 1.25)
	3	-0.05 (-0.19, 0.09)	0.09 (-0.004, 0.18)	0.94 (0.82, 1.90)

All models adjusted for age, sex, education, income and race

^†^All represent comparisons to tertile 1 (reference groups)

### Association between Neighborhood and Dietary Intake

Contrary to our hypothesis, those who lived in poorer neighborhoods had lower intakes of total and saturated fat compared to those living in wealthier neighborhoods (Table 5). This did not appear to be influenced by total caloric intake.

**Table 5:** Adjusted Prevalence Ratios, β coefficients and 95% confidence intervals for Neighborhood Indicators and Dietary Intake among 629 participants in the Look AHEAD Study

Neighborhood Indicator	Tertile	Total calories (kcal) β coefficient (95% CI)	% Total Fat (35 + vs <35)	% Saturated Fat (10 + vs. <10)	Fruit & Vegetable (9 + vs <9 servings)
Below Poverty^† ^(%)	2	56.05 (-123.63, 235.73)	**0.53** **(0.32, 0.87)**	**0.54** **(0.30, 0.98)**	0.84 (0.35, 2.03)
	3	-61.63 (-255.56, 132.30)	**0.57** **(0.33, 0.99)**	0.55 (0.29, 1.06)	1.92 (0.66, 5.61)
					
Food Stores	≥1 vs <1	-86.13 (-234.68, 62.42)	1.02 (0.69, 1.53)	1.28 (0.81, 2.00)	1.34 (0.66, 2.73)
					
Convenience Stores	≥1 vs <1	-0.06 (-146.58, 146.46)	1.02 (0.69, 1.52)	1.18 (0.76, 1.83)	1.64 (0.79, 3.41)
					
Restaurants^†^	2	-63.54 (-236.99, 109.90)	1.31 (0.81, 2.11)	1.00 (0.60, 1.65)	1.26 (0.56, 2.83)
	3	68.86 (-108.78, 246.49)	1.18 (0.73, 1.91)	1.17 (0.67, 2.03)	1.24 (0.52, 2.94)

^†^All represent comparisons to tertile 1 (reference groups)

All models adjusted for age, sex, education, income and race

## Discussion

Our results suggest that among this group of overweight adults with type 2 diabetes in the Look AHEAD study that: 1) the presence of more restaurants in the neighborhood was associated with eating at non-fast food restaurants and with participation in several food and physical activity weight control practices; 2) neighborhood SES was only associated with a few of the weight-related factors. These conclusions are supported by a study with a diverse range of neighborhoods, detailed individual-level data, and a large percentage of minority participants.

Studies of neighborhood and health have generally focused on the physical built environment and its relation to physical activity[35-41]. Some studies have evaluated neighborhood and dietary patterns[42-44], and more recent studies have evaluated obesity or weight status as an outcome [45-50]. Few studies, to date, have evaluated neighborhood SES or other characteristics with weight control practices. Our study, which was conducted within a large-scale weight loss trial, had the strength of including a wealth of individual-level data on eating patterns, weight control practices, along with resource use for weight loss purposes.

There were, however, a few limitations. First, using the census tract as a proxy for neighborhood has been criticized, however, many studies have used this indicator, allowing us to compare our findings across studies. Furthermore, the wealth of data available from the US Census provides a comprehensive view of this geographic entity. Similarly, the neighborhood data may not have represented the entire baseline time-period for the Look AHEAD study. Data used were from the 2000 Census and 2004 Consumer database; Look AHEAD participants were recruited from 2001-2004. Neighborhoods are constantly changing, however the time-frame for the data used was close to the study recruitment period. Second, given the eligibility criteria for entry into the study, the population was fairly homogeneous with respect to some factors. One example was weight, which may explain why there was little variation of BMI status by neighborhood. This may explain many of our negative findings. In a future study, we plan to conduct longitudinal analyses and determine how neighborhood influences response to the weight loss intervention. The longitudinal analyses should show more variation in the dependent variables as individuals respond differently to the intervention. Furthermore, as this was an exploratory study, there were many negative findings that may have been due to low statistical power.

## Conclusion

Future studies should evaluate neighborhood in relation to weight loss behaviors in other populations and further explore the impact of various aspects of the physical and social neighborhood environment. Now that individual-level correlates of healthy weight and weight loss are fairly well understood, attention should be given to other social and environmental determinants that may have a substantial impact. In addition to policy changes such as those that regulate the unhealthy selections in restaurants on an environmental level, incorporating teaching points on influences such as portion control and choosing health options in restaurants should contribute to more successful weight-loss interventions.

## Competing interests

The authors declare that they have no competing interests.

## Authors' contributions

TLG and FLB were responsible for the conception and design of the study. KB was responsible for the data management and data analysis. LP advised on the statistical analysis. TLG drafted the manuscript. KB, JW, JP, XFP, AFB, LJ, FLB were responsible for critical review of the manuscript and interpretation of findings. All authors have read and approved the final manuscript.

## Pre-publication history

The pre-publication history for this paper can be accessed here:

http://www.biomedcentral.com/1471-2458/10/312/prepub

## References

[B1] National Institutes of Health NHLBI Clinical Guidelines on the Identification, Evaluation, and Treatment of Overweight and Obesity in Adults 1998 98 4083 Ref Type: Report 9813653

[B2] Flegal KM Carroll MD Kuczmarski RJ Johnson CL Overweight and obesity in the United States: prevalence and trends, 1960-1994 Int J Obes Relat Metab Disord 1998 22 39 47 10.1038/sj.ijo.0800541 9481598

[B3] Flegal KM Carroll MD Ogden CL Johnson CL Prevalence and trends in obesity among US adults, 1999-2000 JAMA 2002 288 1723 1727 10.1001/jama.288.14.1723 12365955

[B4] Fontaine KR Redden DT Wang C Westfall AO Allison DB Years of life lost due to obesity JAMA 2003 289 187 193 10.1001/jama.289.2.187 12517229

[B5] Flegal KM Carroll MD Kuczmarski RJ Johnson CL Overweight and obesity in the United States: prevalence and trends, 1960-1994 Int J Obes Relat Metab Disord 1998 22 39 47 10.1038/sj.ijo.0800541 9481598

[B6] Faith MS Fontaine KR Baskin ML Allison DB Toward the reduction of population obesity: macrolevel environmental approaches to the problems of food, eating, and obesity Psychol Bull 2007 133 205 226 10.1037/0033-2909.133.2.205 17338597

[B7] Lopez RP Neighborhood risk factors for obesity 1 Obesity (Silver Spring) 2007 15 2111 2119 10.1038/oby.2007.251 17712130

[B8] Papas MA Alberg AJ Ewing R Helzlsouer KJ Gary TL Klassen AC The built environment and obesity 1 Epidemiol Rev 2007 29 129 143 10.1093/epirev/mxm009 17533172

[B9] Cohen DA Mason K Bedimo A Scribner R Basolo V Farley TA Neighborhood physical conditions and health Am J Public Health 2003 93 467 471 10.2105/AJPH.93.3.467 12604497 PMC1447765

[B10] Diez Roux AV Investigating neighborhood and area effects on health Am J Public Health 2001 91 1783 1789 10.2105/AJPH.91.11.1783 11684601 PMC1446876

[B11] Franzini L Caughy M Spears W Fernandez Esquer ME Neighborhood economic conditions, social processes, and self-rated health in low-income neighborhoods in Texas: a multilevel latent variables model 1 Soc Sci Med 2005 61 1135 1150 10.1016/j.socscimed.2005.02.010 15970226

[B12] Diez-Roux AV Nieto FJ Muntaner C Tyroler HA Comstock GW Shahar E Neighborhood environments and coronary heart disease: a multilevel analysis Am J Epidemiol 1997 146 48 63 9215223 10.1093/oxfordjournals.aje.a009191

[B13] Diez-Roux AV Nieto FJ Caulfield L Tyroler HA Watson RL Szklo M Neighbourhood differences in diet: the Atherosclerosis Risk in Communities (ARIC) Study J Epidemiol Community Health 1999 53 55 63 10.1136/jech.53.1.55 10326055 PMC1756776

[B14] Diez Roux AV Merkin SS Arnett D Chambless L Massing M Nieto FJ Neighborhood of residence and incidence of coronary heart disease N Engl J Med 2001 345 99 106 10.1056/NEJM200107123450205 11450679

[B15] Diez Roux AV Jacobs DR Kiefe CI Neighborhood characteristics and components of the insulin resistance syndrome in young adults: the coronary artery risk development in young adults (CARDIA) study Diabetes Care 2002 25 1976 1982 10.2337/diacare.25.11.1976 12401742

[B16] Diez Roux AV Residential environments and cardiovascular risk J Urban Health 2003 80 569 589 14709706 10.1093/jurban/jtg065 PMC3456219

[B17] Henderson C Diez Roux AV Jacobs DR Jr Kiefe CI West D Williams DR Neighbourhood characteristics, individual level socioeconomic factors, and depressive symptoms in young adults: the CARDIA study J Epidemiol Community Health 2005 59 322 328 10.1136/jech.2003.018846 15767387 PMC1733059

[B18] Lisabeth LD Diez Roux AV Escobar JD Smith MA Morgenstern LB Neighborhood environment and risk of ischemic stroke: the brain attack surveillance in Corpus Christi (BASIC) Project Am J Epidemiol 2007 165 279 287 10.1093/aje/kwk005 17077168

[B19] Nordstrom CK Diez Roux AV Jackson SA Gardin JM The association of personal and neighborhood socioeconomic indicators with subclinical cardiovascular disease in an elderly cohort. The cardiovascular health study Soc Sci Med 2004 59 2139 2147 10.1016/j.socscimed.2004.03.017 15351479

[B20] Schootman M Andresen EM Wolinsky FD Malmstrom TK Miller JP Yan Y The effect of adverse housing and neighborhood conditions on the development of diabetes mellitus among middle-aged African Americans Am J Epidemiol 2007 166 379 387 10.1093/aje/kwm190 17625220 PMC4519088

[B21] Schootman M Andresen EM Wolinsky FD Malmstrom TK Miller JP Miller DK Neighbourhood environment and the incidence of depressive symptoms among middle-aged African Americans J Epidemiol Community Health 2007 61 527 532 10.1136/jech.2006.050088 17496262 PMC2465732

[B22] Wang MC Kim S Gonzalez AA MacLeod KE Winkleby MA Socioeconomic and food-related physical characteristics of the neighbourhood environment are associated with body mass index J Epidemiol Community Health 2007 61 491 498 10.1136/jech.2006.051680 17496257 PMC2465719

[B23] Van Lenthe FJ Mackenbach JP Neighbourhood deprivation and overweight: the GLOBE study Int J Obes Relat Metab Disord 2002 26 234 240 10.1038/sj.ijo.0801841 11850756

[B24] Wang MC Kim S Gonzalez AA MacLeod KE Winkleby MA Socioeconomic and food-related physical characteristics of the neighbourhood environment are associated with body mass index J Epidemiol Community Health 2007 61 491 498 10.1136/jech.2006.051680 17496257 PMC2465719

[B25] Tuomilehto J Lindstrom J Eriksson JG Valle TT Hamalainen H Ilanne-Parikka P Prevention of type 2 diabetes mellitus by changes in lifestyle among subjects with impaired glucose tolerance 1 N Engl J Med 2001 344 1343 1350 10.1056/NEJM200105033441801 11333990

[B26] Whelton PK Appel LJ Espeland MA Applegate WB Ettinger WH Jr Kostis JB Sodium reduction and weight loss in the treatment of hypertension in older persons: a randomized controlled trial of nonpharmacologic interventions in the elderly (TONE). TONE Collaborative Research Group 1 JAMA 1998 279 839 846 10.1001/jama.279.11.839 9515998

[B27] Pi-Sunyer X Blackburn G Brancati FL Bray GA Bright R Clark JM Reduction in weight and cardiovascular disease risk factors in individuals with type 2 diabetes: one-year results of the look AHEAD trial 1 Diabetes Care 2007 30 1374 1383 10.2337/dc07-0048 17363746 PMC2665929

[B28] Ryan DH Espeland MA Foster GD Haffner SM Hubbard VS Johnson KC Look AHEAD (Action for Health in Diabetes): design and methods for a clinical trial of weight loss for the prevention of cardiovascular disease in type 2 diabetes Control Clin Trials 2003 24 610 628 10.1016/S0197-2456(03)00064-3 14500058

[B29] Diez Roux AV Jacobs DR Kiefe CI Neighborhood characteristics and components of the insulin resistance syndrome in young adults: the coronary artery risk development in young adults (CARDIA) study Diabetes Care 2002 25 1976 1982 10.2337/diacare.25.11.1976 12401742

[B30] Winkleby MA Cubbin C Influence of individual and neighbourhood socioeconomic status on mortality among black, Mexican-American, and white women and men in the United States J Epidemiol Community Health 2003 57 444 452 10.1136/jech.57.6.444 12775792 PMC1732485

[B31] Krieger N Chen JT Waterman PD Soobader MJ Subramanian SV Carson R Geocoding and monitoring of US socioeconomic inequalities in mortality and cancer incidence: does the choice of area-based measure and geographic level matter?: the Public Health Disparities Geocoding Project Am J Epidemiol 2002 156 471 482 10.1093/aje/kwf068 12196317

[B32] Diez-Roux AV Multilevel analysis in public health research Annu Rev Public Health 2000 21 171 192 10.1146/annurev.publhealth.21.1.171 10884951

[B33] Bingenheimer JB Raudenbush SW Statistical and substantive inferences in public health: issues in the application of multilevel models Annu Rev Public Health 2004 25 53 77 10.1146/annurev.publhealth.25.050503.153925 15015912

[B34] Klassen AC Curriero FC Hong JH Williams C Kulldorff M Meissner HI The role of area-level influences on prostate cancer grade and stage at diagnosis Prev Med 2004 39 441 448 10.1016/j.ypmed.2004.04.031 15313082

[B35] Humpel N Owen N Leslie E Environmental factors associated with adults' participation in physical activity: a review Am J Prev Med 2002 22 188 199 10.1016/S0749-3797(01)00426-3 11897464

[B36] Addy CL Wilson DK Kirtland KA Ainsworth BE Sharpe P Kimsey D Associations of perceived social and physical environmental supports with physical activity and walking behavior Am J Public Health 2004 94 440 443 10.2105/AJPH.94.3.440 14998810 PMC1448272

[B37] Suminski RR Poston WS Petosa RL Stevens E Katzenmoyer LM Features of the neighborhood environment and walking by U.S. adults Am J Prev Med 2005 28 149 155 10.1016/j.amepre.2004.09.009 15710269

[B38] Moudon AV Lee C Cheadle AD Garvin C Rd DB Schmid TL Attributes of environments supporting walking Am J Health Promot 2007 21 448 459 17515010 10.4278/0890-1171-21.5.448

[B39] Librett JJ Yore MM Schmid TL Kohl HW III Are self-reported physical activity levels associated with perceived desirability of activity-friendly communities? Health Place 2007 13 767 773 10.1016/j.healthplace.2006.07.003 16935021

[B40] Cohen DA McKenzie TL Sehgal A Williamson S Golinelli D Lurie N Contribution of public parks to physical activity Am J Public Health 2007 97 509 514 10.2105/AJPH.2005.072447 17267728 PMC1805017

[B41] Diez Roux AV Evenson KR McGinn AP Brown DG Moore L Brines S Availability of recreational resources and physical activity in adults Am J Public Health 2007 97 493 499 10.2105/AJPH.2006.087734 17267710 PMC1805019

[B42] Cheadle A Psaty BM Curry S Wagner E Diehr P Koepsell T Community-level comparisons between the grocery store environment and individual dietary practices Prev Med 1991 20 250 261 10.1016/0091-7435(91)90024-X 2057471

[B43] Morland K Wing S Diez RA The contextual effect of the local food environment on residents' diets: the atherosclerosis risk in communities study Am J Public Health 2002 92 1761 1767 10.2105/AJPH.92.11.1761 12406805 PMC1447325

[B44] Moore LV Diez Roux AV Associations of neighborhood characteristics with the location and type of food stores Am J Public Health 2006 96 325 331 10.2105/AJPH.2004.058040 16380567 PMC1470485

[B45] Inagami S Cohen DA Finch BK Asch SM You are where you shop: grocery store locations, weight, and neighborhoods Am J Prev Med 2006 31 10 17 10.1016/j.amepre.2006.03.019 16777537

[B46] Glass TA Rasmussen MD Schwartz BS Neighborhoods and obesity in older adults: the Baltimore Memory Study Am J Prev Med 2006 31 455 463 10.1016/j.amepre.2006.07.028 17169707 PMC1851911

[B47] Boehmer TK Hoehner CM Deshpande AD Brennan Ramirez LK Brownson RC Perceived and observed neighborhood indicators of obesity among urban adults Int J Obes (Lond) 2007 in press 17224932 10.1038/sj.ijo.0803531

[B48] Rundle A Roux AV Free LM Miller D Neckerman KM Weiss CC The urban built environment and obesity in New York City: a multilevel analysis Am J Health Promot 2007 21 326 334 17465178 10.4278/0890-1171-21.4s.326

[B49] Wang MC Kim S Gonzalez AA MacLeod KE Winkleby MA Socioeconomic and food-related physical characteristics of the neighbourhood environment are associated with body mass index J Epidemiol Community Health 2007 61 491 498 10.1136/jech.2006.051680 17496257 PMC2465719

[B50] Papas MA Alberg AJ Ewing R Helzlsouer KJ Gary TL Klassen AC The built environment and obesity 1 Epidemiol Rev 2007 29 129 143 10.1093/epirev/mxm009 17533172

